# Parental History of Major Depressive Disorder Moderates the Relation Between Neighborhood Disadvantage and Reward Responsiveness in Children

**DOI:** 10.1007/s10802-025-01310-4

**Published:** 2025-03-22

**Authors:** Elana S. Israel, Cope Feurer, Aliona Tsypes, Brandon E. Gibb

**Affiliations:** 1 Department of Psychology, Binghamton University (SUNY), Binghamton, USA; 2 Department of Psychiatry, University of North Carolina at Chapel Hill, Chapel Hill, USA; 3 Department of Psychiatry, University of Pittsburgh, Pittsburgh, USA

**Keywords:** Neighborhood disadvantage, Reward positivity, Event-related potentials, Parental depression

## Abstract

In this study, we examined associations between census-derived indices of neighborhood disadvantage and children’s reward outcome processing and whether these relations would be stronger among children already at risk for alterations in reward processing due to having a parental history of major depressive disorder (MDD) compared to children of never depressed parents. Participants were 224 children aged 7–11 years old and their parent. Parents were required to either have a history of MDD or no lifetime history of any depressive disorder. To measure reward outcome processing, we focused on the reward positivity (RewP) event-related potential (ERP) elicited following gain and loss outcome feedback while children completed a monetary reward task. Census-derived measures of neighborhood disadvantage based upon families’ addresses included the Area Deprivation Index (ADI), neighborhood crime risk, and the Child Opportunity Index (COI). The general pattern of findings across indices was that higher levels of neighborhood disadvantage were associated with more blunted neural reactivity to both gain and loss feedback, but only among children with a parental history of MDD and not among children of never depressed parents. These results suggest that broader contextual stressors may impact how youth process reward outcome feedback, especially youth already at heightened risk for depression, which may have implications for understanding risk for disorders associated with reward dysfunction.

There is growing evidence that neighborhood socioeconomic disadvantage is associated with detrimental mental health outcomes among youth, which may be due, in part, to greater exposure to environmental stressors (Sabel et al., 2024; Wadsworth et al., 2016; Xue et al., 2005). Theorists have suggested that exposure to stress impacts the function of the mesolimbic dopaminergic system, which can affect how individuals process reward in their environment (Auerbach et al., 2014; Baik, 2020). Supporting this, research using both human and animal models has documented the adverse effects that acute stressors and repeated stress exposure during development have on neural reward circuitry (Dillon et al., 2014; Novick et al., 2018; Sheth et al., 2017; Watt et al., 2017). However, previous research has largely focused on individual-level stressors and less is known about how chronic, contextual stressors, like neighborhood disadvantage, influence the development of reward processing in youth. Investigating how neighborhood disadvantage relates to youth’s reward processing is important because it can (i) help to identify aspects of one’s broader environment that may heighten risk for reward dysfunction and (ii) help to target communities of risk that may benefit from intervention efforts aimed at reducing rates of emotional and behavioral disorders associated with reward dysfunction in youth.

To date, studies examining links between socioeconomic/neighborhood variables and reward processing in youth have yielded mixed results, with some but not all studies supporting the relation (see, e.g., Chat et al., 2022; Gonzalez et al., 2015; Granros et al., 2024; Jorgensen et al., 2022; Mullins et al., 2020; Romens et al., 2015; White et al., 2022) suggesting that there may be important moderators of risk. Consistent with vulnerability-stress models, Granros et al. (2024) recently showed that a family history of major depressive disorder (MDD) moderated the link between neighborhood disadvantage and reward processing deficits in youth. Specifically, using the reward positivity (RewP^1^) event-related potential (ERP) component to index initial neural responsiveness to reward outcome feedback (monetary gain versus loss; see Glazer et al., 2018; Proudfit, 2015), Granros et al. (2024) showed that the link between a census-derived index of neighborhood disadvantage (the Area Deprivation Index; ADI) and blunted RewP was only observed among youth with a maternal history of MDD and not among offspring of never depressed mothers. This supports a vulnerability-stress hypothesis, in that youth with a parental MDD history may be more vulnerable to the effects of contextual stress on alterations in reward processing.

The goal of this study was to replicate and extend the findings presented by Granros et al. (2024). In assessing neighborhood disadvantage, Granros et al. (2024) focused on the ADI, which is a broad index of socioeconomic disadvantage (incorporating factors like housing quality, income, employment, and education). Supporting the importance this broad measure of disadvantage, higher ADI levels are associated with higher levels of internalizing and externalizing symptoms in youth (Beyer et al., 2024; Ip et al., 2022) However, neighborhood disadvantage is a heterogeneous construct that includes exposure to community violence (threat) and reduced access to resources (deprivation) and these two forms of disadvantage may have differential impacts on child brain development and, in turn, risk for the development of psychopathology (Hyde et al., 2022; McLaughlin et al., 2014). Previous research shows that both threat and deprivation are associated with internalizing and externalizing symptoms in youth (Miller et al., 2021; Sadikova et al., 2024; Schäfer et al., 2023; Wang et al., 2022) as well as alterations in youth’s reward functioning (Hein et al., 2020; Sadikova et al., 2024). However, few studies have examined whether objective measures of threat (e.g., crime rates) and deprivation (e.g., ADI levels) relate to neural reward processing and the extant research is mixed as to whether these variables are predictive of youth’s reward functioning (Chat et al., 2022; Granros et al., 2024; Mullins et al., 2020). Thus, it is important to examine whether objective indices of both threat and deprivation relate to reward processing in children who are already at heightened risk for disorders related to alterations in reward processing.

This current study, therefore, examined whether objective measures of neighborhood deprivation and threat were linked to neural measures of reward processing in children at high risk of developing depression. Specifically, in addition to the ADI, we also included census-derived indices of neighborhood crime risk (reflecting levels of threat) as well as levels of opportunity available to children in a given community (the Child Opportunity Index [COI]; reflecting levels of deprivation). We hypothesized that higher levels of neighborhood disadvantage (higher levels of socioeconomic disadvantage and crime and lower levels of opportunity) would be associated with more blunted reward responsiveness. We also hypothesized that these relations would be stronger among children of parents who had a history of MDD than among children of never depressed parents. Finally, given evidence from prior studies of relations between reward processing and both socioeconomic status/family income (Romens et al., 2015; White et al., 2022) and symptoms of depression (Luking et al., 2016; Thompson et al., 2023) in youth, we conducted sensitivity analyses to determine whether any significant relations would be maintained after statistically controlling for the influence of these variables as well as relevant demographic variables (children’s age, sex, and race/ethnicity).

## Method

### Participants

Participants were 250 children aged 7–11 years old, and their participating parent, recruited from the community between January 2013 and September 2016 through social media and television advertisements, billboards, and flyers. This age range was selected so that we could examine potential early contributors to risk for disorders that tend to increase during adolescence (e.g., depression and substance misuse). If there was more than one caregiver in the household, the parent who contacted the researchers was the participating parent. Participating parents either had a lifetime history of major depressive disorder (MDD) or no lifetime history of MDD. Parents who had a lifetime history of bipolar disorder or a psychotic disorder were excluded. The only exclusion criterion for children was the presence of a learning disability or developmental disorder, reported by parents, that could affect the child’s ability to complete the study. Of the original 250 children, 26 were excluded because of equipment failure or having insufficient data during the monetary reward task. Thus, the final sample was 224 parent-child dyads.

The mean age of the children included in the final sample was 9.68 (*SD* = 1.41) and 46.88% were female. In terms of race, 74.11% of the children were White, 13.84% were Black/African American, 11.16% were more than one race, 0.45% were Asian American and, in terms of ethnicity, 9.82% were Hispanic. Of the parents, 86.61% were biological mothers, 11.16% were biological fathers, and the remaining 2.23% were other caretakers (4 female, 1 male). The average age of parents in this sample was 37.99 (*SD* = 6.28). In terms of race, 82.14% of the parents were White, 15.18% were Black/African American, 1.34% were Asian American, 1.34% were more than one race, and, in terms of ethnicity, 5.36% were Hispanic. The median family income was $40,001-$45,000.

### Procedure

Upon arrival to the laboratory, parents were asked to provide informed consent and children were asked to provide assent. Parents then completed a diagnostic interview and children completed the Doors Task while electroencephalography (EEG) data were collected. Parents provided their current address so that we could derive indices of neighborhood disadvantage for each family. Families were compensated monetarily for their participation. All study procedures were approved by the Binghamton University Institutional Review Board.

### Measures

#### Parent History of MDD

Parents’ history of MDD was assessed using the Structured Clinical Interview for DSM-IV-TR Axis I Disorders (SCID; First et al., 2002). A subset of 20 interviews were coded by a second diagnostician and interrater reliability for diagnoses of MDD was good (κ = 0.89). Of the parents, 50.00% met criteria for past or current MDD and the remainder had no lifetime history of MDD. Of the parents who had a history of MDD, 8.93% were in a current episode of MDD at the time of the assessment.

#### Neighborhood Indices

##### Neighborhood Definition

Participants’ neighborhoods were determined based on the zip code of their current address at the time of study enrollment.

##### Area Deprivation Index

Neighborhood socioeconomic disadvantage indices were obtained from the 2015 Area Deprivation Index (ADI; Kind & Buckingham, 2018). The ADI is a factor score calculated from 5-year census data from the American Community Survey on 10 socioeconomic factors including income, education, employment, and housing quality. The ADI provides a nationally-ranked index score ranging from 1 to 100, such that a score of 1 indexes the lowest levels of area disadvantage compared to the national average and a score of 100 indicates the highest levels of disadvantage.

The ADI defines neighborhoods as census block groups, which is consistent with prior literature in large urban areas. However, examinations of area socioeconomic deprivation in rural and suburban areas remain sparse, and there is a lack of consensus regarding the extent to which census block groups appropriately capture neighborhoods outside of urban areas (de Marco & de Marco, 2010). Furthermore, as census block groups are defined based on population size, their geospatial size tends to be much larger in rural areas compared to suburban and urban areas, thereby making comparison of neighborhoods difficult when considering studies encompassing both rural and non-rural areas, like in the current study. Therefore, to be consistent with prior research using the current sample, neighborhoods were defined by participants’ zip codes. Consistent with prior studies that have examined the ADI at the zip code level (Chan et al., 2021; Liu et al., 2023), all ADI indices within participant zip codes were averaged. In the current study, participants’ ADI scores ranged from 41 to 97 (*M* = 71.75, *SD* = 10.51) with a higher score reflecting greater neighborhood disadvantage.

##### Neighborhood Crime

The neighborhood crime risk index was obtained from the 2015 CrimeRisk database (Applied Geographic Solutions, 2015), which is a database containing geocoded information about crime risk across various types of crime including property (i.e., burglary, larceny, motor vehicle theft) and personal (i.e., murder, rape, robbery, assault) crime rates for each zip code within the target county. A crime risk index, indicating the relative risk of a crime occurring in an area compared to the national average, was derived from a thorough analysis of crime reports in the target county across a 7-year period. A score of “100” reflects the national average for crime risk. In the current study, crime risk ranged from 13 to 143 (*M* = 81.05, *SD* = 36.54).^2^

##### Child Opportunity Index

Indices of neighborhood opportunity were obtained from the 2015 Childhood Opportunity Index 2.0 (COI; Acevedo-Garcia et al., 2014). The COI is calculated using publicly available data from a variety of sources (e.g., the American Community Survey, the Department of Education, the Environmental Protection Agency) aggregated at the zip code level to provide nationally-normed indices of the quality of resource availability and opportunities within one’s neighborhood. The COI is calculated for both overall neighborhood opportunity and within three specific sub-domains: education (e.g., number of high-quality early childhood education centers, advanced placement course enrollment, teacher experience), health and environment (e.g., access to healthy food, health insurance coverage, toxin exposure), and social and economic (e.g., employment rate, public assistance rate, work commute). In the current study, COI composite scores ranged from 4 to 94 (*M* = 45.58, *SD* = 23.43), the education subscale scores ranged from 2 to 94 (*M* = 46.45, *SD* = 23.55), the health and environment subscale scores ranged from 35 to 98 (*M* = 66.95, *SD* = 19.82), and the social and economic subscale scores ranged from 3 to 98 (*M* = 41.56, *SD* = 23.81).

#### Reward Processing

##### Reward Task

Reward processing was measured using the Doors Task, which is commonly used in studies examining reward processing in youth (Proudfit, 2015). Each trial began with the presentation of two doors, one on the left and one on the right side of the screen. Participants were told to guess which door has a prize behind it and that they would get $0.50 for each correct guess but lose $0.25 for each incorrect guess. The doors remained on the screen until the participants made their choice using a game controller (Logitech F310). Following the behavioral response, a fixation cross was presented for 1000 ms. After this, participants received feedback that they had either won $0.50 (green up-arrow) or lost $0.25 (red down-arrow) on that round. This feedback slide remained on the screen for 2000 ms. Following feedback, the participants were instructed to click either button on the game controller to proceed to the next round, with a randomly jittered inter-trial interval of 400–650 ms after they clicked to proceed. The task contained 50 trials, consisting of 25 gain and 25 loss trials presented in a random order in two blocks of 25 trials. All participants received $5 immediately after completing the task.

##### EEG Data Acquisition and Processing

Continuous EEG was recorded during the Doors Task using a BioSemi ActiveTwo system and was digitized at a 24-bit resolution with a sampling rate of 512 Hz. EEG recordings were taken from 34 scalp electrodes: 32 electrodes based on the 10/20 system and two additional (FCz and Iz). Electrooculogram was recorded from four electrodes placed above and below the right eye and on the outer canthus of each eye to aid in inspection of vertical and horizontal eye movements in the EEG time series.

We performed offline analysis using the MATLAB extension EEGLAB (Delorme & Makeig, 2004) and the EEGLAB plug-in ERPLAB (Lopez-Calderon & Luck, 2014). EEG data were re-referenced to the average of the left and right mastoid electrodes and the DC was removed by subtracting the mean value of the EEG signal. EEG data were band-passed filtered with cut-offs of 0.1 and 30 Hz using a second-order IIR Butterworth filter with a 12 dB/octave roll-off. The data were then processed to reject and correct artifacts. Independent component analysis (ICA) was used to identify and remove large and stereotypical ocular components by inspecting scalp maps (Jung et al., 2001). EEG was segmented into epochs for each trial beginning 200 ms before the onset of the feedback stimulus and ending 1000 ms after the onset of the feedback stimulus. Epochs that contained large artifacts (> 100 µV) were removed before analyses. Youth were included in the analyses if they had at least 14 trials per gain/loss condition, based on previous work in youth samples showing that researchers could obtain a reliable neural signal from the Doors Task with as few as 14 trials in each condition (Luking et al., 2017). The average number of gain trials following artifact rejection was 23.38 (*SD* = 2.89) and the average number of loss trials was 23.09 (*SD* = 3.09).

##### PCA

As in our previous studies (e.g., Gibb et al., 2022), we used temporospatial principal component analysis (PCA; Dien, 2010b) to isolate neural activity consistent with the characteristics of the RewP-gain and RewP-loss. We choose this approach because the RewP is one of several temporally and spatially overlapping subcomponents of reward-outcome processing that unfold quickly over time (Glazer et al., 2018). In these types of situations, PCA may provide a “purer” measure of a specific ERP than traditional mean amplitude-based approaches (Dien, 2012).

**Fig. 1:**
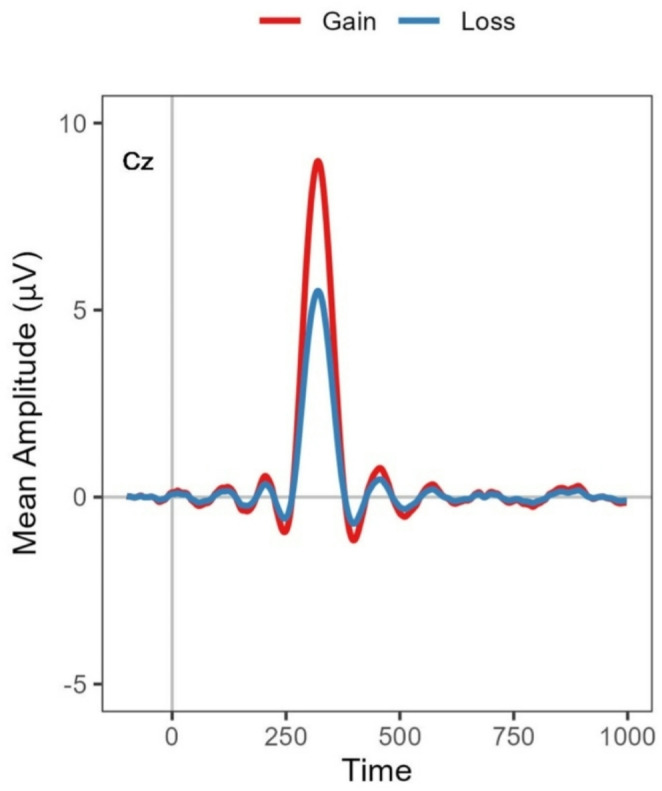
PCA-derived RewP waveform

PCA was conducted using the ERP PCA Toolkit, version 2.92 (Dien, 2010b) and used the guidelines published for the use of PCA with ERP data (Dien, 2010a). First, a temporal PCA was performed using a temporal Promax rotation to rotate the structure in the temporal domain. The temporal PCA incorporated the millisecond-level timepoints within each trial as variables and included all participants, both conditions, and the 34 electrode cites as observations. Nineteen temporal factors were extracted for rotation based on a parallel test (Horn, 1965) of the Scree plot (Cattell, 1966). Following the temporal PCA, a spatial PCA was performed using a spatial Infomax rotation that was conducted on each temporal factor. For the spatial PCA, electrode sites were used as variables and participants, conditions, and temporal factor scores were used as observations. Three spatial factors were extracted from each temporal factor based on the results of a parallel test, which resulted in 57 temporospatial factor combinations that accounted for 79.92% of the variance in the data. For this study, we focused on factors that matched the known temporal and spatial characteristics of the RewP (TF5/SF1). For further details of the PCA analyses, see Gibb et al. (2022). The PCA-derived RewP waveform can be found in Fig. 1.

##### Mean Amplitude-Based ERP

**Fig. 2:**
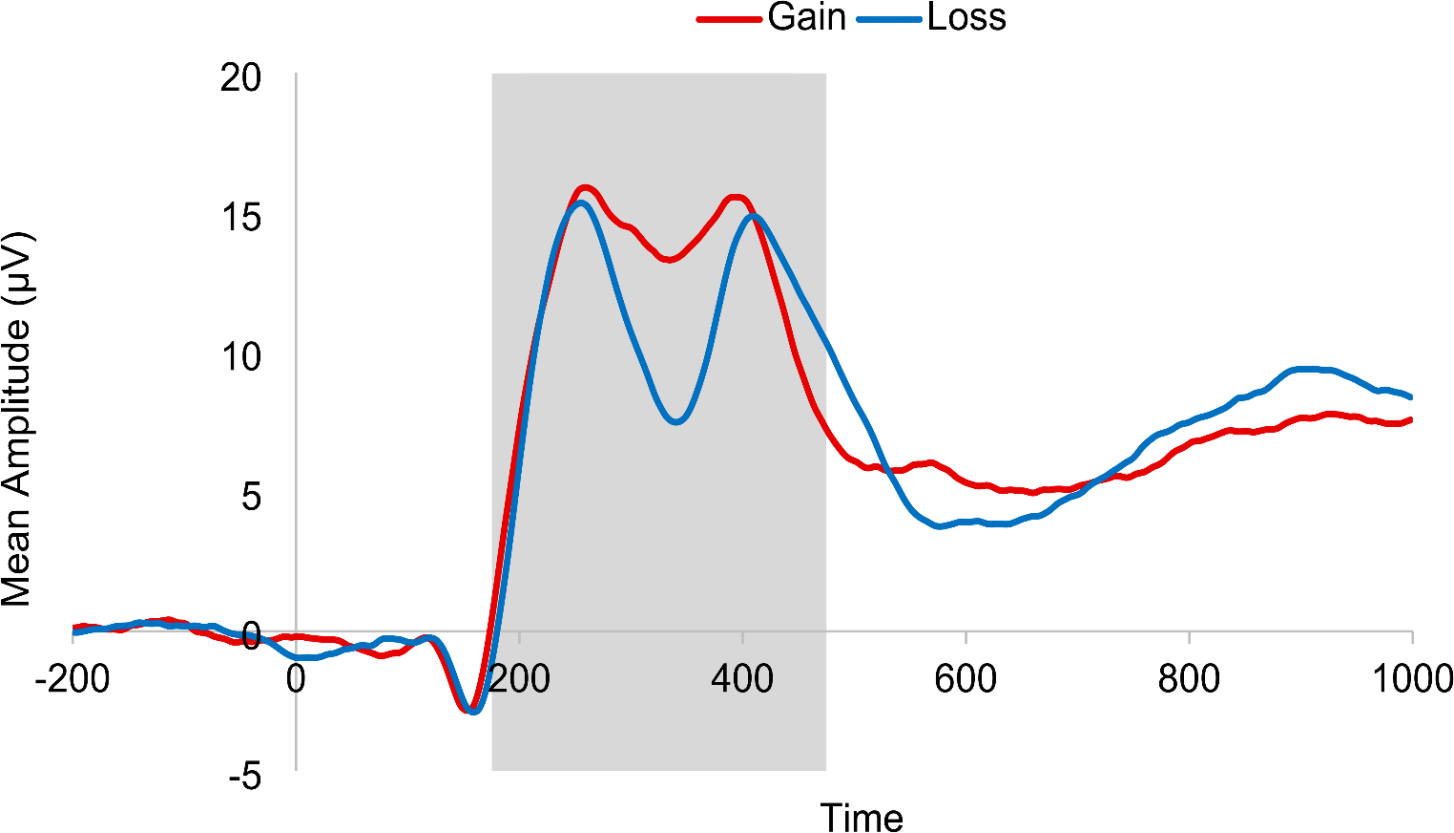
Mean amplitude-based RewP waveform

Although our primary analyses focused on a PCA derived temporospatial factor that was characteristic of the RewP, we repeated our analyses using a mean amplitude-based ERP, which is a more traditional method of deriving ERP components. The mean amplitude-based RewP was the average amplitude 275–375 milliseconds after gain and loss feedback was presented at FCz. The mean amplitude-based RewP waveform can be found in Fig. 2.

#### Children’s Symptoms of Depression

The Children’s Depression Inventory (CDI; Kovacs, 1981) was used to measure youth depression symptoms. It is a 27-item self-report questionnaire that is used with youth ages 7–17 years old. The CDI has been shown to have strong reliability and validity when used in youth community samples (e.g., Smucker et al., 1986). The CDI demonstrated good internal consistency in the current sample (α = 0.80).

### Analysis Plan

The goal of this study was to examine links between indices of neighborhood disadvantage and children’s reward outcome processing (RewP-gain and RewP-loss) and whether these links are moderated by parents’ history of MDD. To test our hypotheses, we used a series of repeated measures general linear models (GLMs) with outcome (RewP-gain vs. RewP-loss) entered as a within-subject factor and parent MDD history, neighborhood index, and the parent MDD × neighborhood index interaction entered as between-subjects variables. Significant interactions were followed by post hoc tests (correlations) to determine the pattern of the interaction. Each of the neighborhood indices (ADI, crime risk, and COI) was examined in a separate analysis. Although, as noted above, we primarily focused on the RewP-gain and RewP-loss derived from PCA, we also examined the mean amplitude-based RewP-gain and RewP-loss to facilitate comparisons with earlier studies.

## Results

A preliminary inspection of the data revealed some missing data (CDI: 4.5%, crime risk: 3.1%, COI: 1.8%, family income: 1.8%). Given this, we examined the pattern of missing data to determine whether estimation of missing values was justified (cf. Schafer & Graham, 2002).^3^ Little’s missing completely at random test, for which the null hypothesis is that the data are missing completely at random (Little & Rubin, 1987), was nonsignificant, *χ*^2^(113) = 96.57, *p* =.87, providing support for the imputation of missing values. Therefore, expectation maximization was used to estimate missing values, which were used in all subsequent analyses (see Schafer & Graham, 2002). Correlations among study variables can be found in Table 1.

**Table 1:** Correlations among study variables

	1	2	3	4	5	6	7	8	9	10	11	12
1. Parent MDD History	-											
2. ADI	0.15	-										
3. Crime Risk	− 0.01	0.76	-									
4. COI-Overall	− 0.10	− 0.87	− 0.60	-								
5. COI-Social/Econ	− 0.11	− 0.89	− 0.66	0.98	-							
6. COI-Education	− 0.14	− 0.71	− 0.37	0.87	0.77	-						
7. COI-Health/Env	0.10	− 0.50	− 0.45	0.71	0.66	0.48	-					
8. PCA RewP-Gain	− 0.10	− 0.14	− 0.09	0.13	0.14	0.10	0.06	-				
9. PCA RewP-Loss	− 0.19	− 0.17	− 0.14	0.12	0.15	0.05	0.02	0.47	-			
10. Mean Amp. RewP-Gain	− 0.18	− 0.11	− 0.12	0.07	0.09	0.03	0.03	0.71	0.38	-		
11. Mean Amp. RewP-Loss	− 0.23	− 0.09	− 0.08	0.03	0.04	0.00	− 0.02	0.45	0.68	0.70	-	
12. CDI	0.03	− 0.06	− 0.05	0.01	0.01	− 0.01	0.05	− 0.04	− 0.01	− 0.01	− 0.06	-
13. Family Income	− 0.12	− 0.23	− 0.16	0.23	0.22	0.20	0.17	0.07	− 0.03	0.02	− 0.02	− 0.14

*Note* MDD = Major depressive disorder. ADI = Area Deprivation Index. COI = Child Opportunity Index. PCA = Principal components analysis. CDI = Children’s Depression Inventory

Correlations *≥*|0.14| significant at *p* <.05. Correlations *≥*|0.18| significant at *p* <.01. Correlations *≥*|0.22| significant at *p* <.001

### PCA-Derived ERPs

A summary of these analyses is presented in Table 2. As can be seen in the table, although the main effect of feedback type was significant in all analyses, there were no significant interactions of feedback type with parental MDD or neighborhood adversity in any of the analyses, indicating that relations observed for parental MDD and/or neighborhood adversity were for responses to outcome feedback generally rather than for feedback regarding gain versus loss specifically.

**Table 2:** Summary of analyses predicting PCA-derived RewP amplitudes

	ADI			Crime risk			COI		
	*F*	*p*	*n*_*p*_^2^	*F*	*p*	*n*_*p*_^2^	*F*	*p*	*n*_*p*_^2^
Parent History of MDD	4.91	0.03	0.02	3.03	0.08	0.02	5.57	0.02	0.03
Neighborhood Index	8.74	0.00	0.04	2.54	0.11	0.01	6.09	0.01	0.03
Feedback Type	44.64	< 0.001	0.17	34.94	< 0.001	0.16	45.66	< 0.001	0.17
Parent MDD × Neigh. Index	12.84	< 0.001	0.06	6.94	0.01	0.04	7.07	0.01	0.03
Parent MDD × Feedback	1.54	0.22	0.01	2.14	0.15	0.01	1.76	0.19	0.01
Neigh. Index × Feedback	0.07	0.79	0.00	0.51	0.48	0.00	0.15	0.70	0.00
Parent MDD × Neigh. Index × Feedback	0.04	0.84	0.00	0.00	0.99	0.00	0.39	0.53	0.00

*Note* MDD = Major depressive disorder. ADI = Area Deprivation Index. COI = Child Opportunity Index

Focusing first on the ADI, there was a significant main effect of parental MDD, *F*(1, 220) = 4.91, *p* =.03, *η*_*p*_^*2*^ = 0.02, with children of parents with a history of MDD exhibiting a significantly smaller RewP collapsed across gain and loss feedback (RewP-gain/loss) than children of never depressed parents. There was also a significant main effect of ADI, *F*(1, 220) = 8.74, *p* =.003, *η*_*p*_^*2*^ = 0.04, with higher ADI scores associated with a smaller RewP response, again collapsing across feedback type. Importantly, the parental MDD × ADI interaction was also significant, *F*(1, 220) = 12.84, *p* <.001, *η*_*p*_^*2*^ = 0.06. Examining the form of this interaction, we found that higher ADI scores were associated with a smaller RewP-gain/loss among children of parents with a history of MDD, *r* = −.39, *p* <.001, but not among children of never depressed parents, *r* =.05, *p* =.64.

Focusing next on neighborhood crime risk, although the main effect of crime risk was not significant, *F*(1, 181) = 2.54, *p* =.11, *η*_*p*_^*2*^ = 0.01, there was a significant parental MDD × crime risk interaction, *F*(1, 181) = 6.94, *p* =.01, *η*_*p*_^*2*^ = 0.04. Examining the form of this interaction, we found that living in a neighborhood characterized by higher crime risk was associated with a more blunted RewP-gain/loss (again, collapsing across gain and loss outcome feedback) among children of parents with a history of MDD, *r* = −.32, *p* =.002, but not among children of never depressed parents, *r* =.07, *p* = 50.^4^

We then examined levels of childhood opportunity, both in terms of overall COI and also the specific subcomponents. There was a significant main effect of overall COI, *F*(1, 220) = 6.09, *p* =.01, *η*_*p*_^*2*^ = 0.03, where lower levels of overall neighborhood opportunity were associated with a more blunted RewP-gain/loss. There was also a significant parental MDD × overall COI interaction, *F*(1, 220) = 7.07, *p* =.01, *η*_*p*_^*2*^ = 0.03, with follow-up tests showing that living in a neighborhood with lower opportunity was associated with a smaller RewP-gain/loss among children of parents with a history of MDD, *r* =.31, *p* =.001, but not children of never depressed parents, *r* = −.01, *p* =.88. Turning next to the individual COI subscales, we found a significant main effect for the social/economic opportunity domain (COI-SE), *F*(1, 220) = 7.50, *p* =.007, *η*_*p*_^*2*^ = 0.03, with lower COI-SE associated with a smaller RewP-gain/loss. We also found a significant parental MDD × COI-SE interaction, *F*(1, 220) = 8.79, *p* =.003, *η*_*p*_^*2*^ = 0.04, such that lower social/economic opportunity was associated with a smaller RewP-gain/loss among children of parents with a history of MDD, *r* =.34, *p* <.001, but not among children of never depressed parents, *r* = −.02, *p* =.86. In contrast, there were no significant main effects or interactions for the education and health/environment subscales (lowest *p* =.14).

**Fig. 3:**
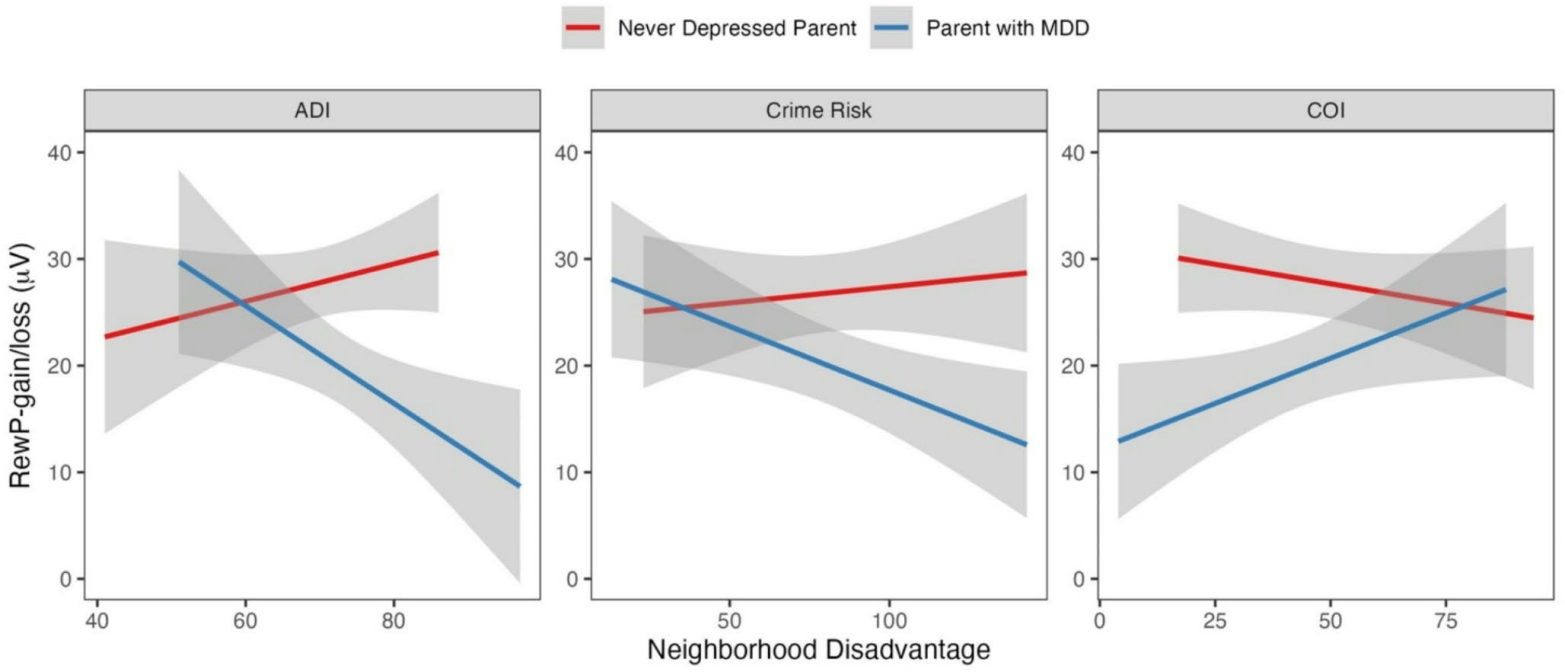
Parental MDD history moderating the association between neighborhood disadvantage variables (ADI, crime risk, and COI) and RewP-gain/loss amplitudes

In summary, therefore, higher levels of neighborhood disadvantage – higher levels of ADI and crime risk and lower levels of COI (overall COI and COI-SE) – were associated with blunted RewP responses to both gain and loss feedback, but only among children of parents with a history of MDD, and not among children of never depressed parents (see Fig. 3). To examine the robustness of these results, we conducted sensitivity analyses to determine if the findings would be maintained even after accounting for other variables known to be associated with reward processing (i.e., child age, sex, race/ethnicity, family income, and depression symptoms).^5^ All of the results remained significant (all *p*s *≤* 0.05).

### Mean Amplitude-Based ERPs

Results from the amplitude-based ERP analyses can be found in Table 3. The pattern of results for these analyses was similar to that described above. Consistent with the PCA-derived ERPs, neither parent MDD history nor any index of neighborhood disadvantage significantly interacted with feedback type (gain vs. loss), indicating that links between our predictor variables and RewP amplitudes were similar for neural responses to both gain and loss feedback.

**Table 3:** Summary of analyses predicting mean amplitude-based rewps

	ADI			Crime risk			COI		
	*F*	*p*	*n*_*p*_^2^	*F*	*p*	*n*_*p*_^2^	*F*	*p*	*n*_*p*_^2^
Parental History of MDD	10.81	0.001	0.05	7.05	0.01	0.04	11.34	< 0.001	0.05
Neighborhood Index	1.50	0.22	0.01	1.56	0.21	0.01	0.86	0.36	0.00
Feedback Type	63.68	< 0.001	0.22	47.08	< 0.001	0.21	64.34	< 0.001	0.23
Parental MDD × Neigh. Index	7.53	0.01	0.03	4.41	0.04	0.02	5.40	0.02	0.02
Parental MDD × Feedback	1.73	0.19	0.01	0.69	0.41	0.00	1.75	0.19	0.01
Neigh. Index × Feedback	0.71	0.40	0.00	0.36	0.55	0.00	1.38	0.24	0.01
Parental MDD × Neigh. Index × Feedback	0.99	0.32	0.00	0.58	0.45	0.00	1.49	0.22	0.01

*Note* MDD = Major depressive disorder. ADI = Area Deprivation Index. COI = Child Opportunity Index

For analyses with the ADI, there was a significant main effect of parental MDD history, *F*(1, 220) = 10.81, *p* =.001, *η*_*p*_^*2*^ = 0.05, where children of parents with a history of MDD had a smaller RewP-gain/loss than children of never depressed parents. Although the main effect of the ADI on RewP amplitudes was not significant *F*(1, 220) = 1.50, *p* =.22, *η*_*p*_^*2*^ = 0.01, there was a significant parental MDD × ADI interaction, *F*(1, 220) = 7.53, *p* =.01, *η*_*p*_^*2*^ = 0.03, such that higher ADI levels were associated with a smaller RewP-gain/loss among children of parents with a history of MDD, *r* = −.24, *p* =.01, but not among children of never depressed parents, *r* =.11, *p* =.23.

Turning next to neighborhood crime risk, the main effect of crime risk on the amplitude-based RewP-gain/loss was not significant, *F*(1, 181) = 1.56, *p* =.21, *η*_*p*_^*2*^ = 0.01. However, the parental MDD × crime risk interaction was significant, *F*(1, 181) = 4.41, *p* =.04, *η*_*p*_^*2*^ = 0.02, with greater crime risk associated with a smaller RewP-gain/loss among children of parents with a history of MDD, *r* = −.25, *p* =.01, but not among children of never depressed parents, *r* =.06, *p* =.57.^6^

Regarding the COI, none of the main effects for the overall COI or the COI subscales were significant. However, there was a significant parental MDD history × overall COI interaction, *F*(1, 220) = 5.40, *p* =.02, *η*_*p*_^*2*^ = 0.02, such that lower COI levels were associated with a smaller RewP-gain/loss among children with a parental MDD history, *r* =.19, *p* =.04, but not among children of never depressed parents, *r* = −.11, *p* =.26. There was also a significant parental MDD × COI-SE interaction, *F*(1, 220) = 6.21, *p* =.01, *η*_*p*_^*2*^ = 0.03, where lower COI-SE levels were associated with smaller RewP-gain/loss amplitudes among children of parents with a history of MDD, *r* =.22, *p* =.02, but not among children with no parental MDD history, *r* = −.10, *p* =.28.

Consistent with the PCA analyses, higher levels of neighborhood disadvantage – higher levels of ADI and crime risk and lower levels of COI (overall COI and COI-SE) – were associated with blunted RewP responses to both gain and loss feedback, but only among children of parents with a history of MDD, and not among children of never depressed parents. Examining the robustness of these results, we conducted sensitivity analyses to determine if our results were maintained when statistically accounting for children’s age, sex, race/ethnicity, family income, and depression symptoms and found that all of the results remained significant (all *p*s *≤* 0.05).

## Discussion

The goal of this study was to replicate and extend prior research showing that the link between neighborhood disadvantage and alterations in youth neural reward outcome processing is stronger for youth who are already at elevated risk for depression based on having a parental history of MDD (Granros et al., 2024). Specifically, we sought to (i) determine whether similar results would be observed in children residing in a different area of the country (upstate New York vs. Chicago in the original study) and (ii) expand the assessment of neighborhood disadvantage to determine whether components of disadvantage focused on threat (e.g., crime risk) versus deprivation (e.g., lack of neighborhood opportunity) may be more strongly related to children’s reward processing. In doing so, we examined whether three indices of contextual stress – neighborhood disadvantage, crime risk, and (lack of) childhood opportunities – were associated with ERP indices of children’s reward outcome processing, and whether these relations would be stronger among children already at risk for blunted reward processing due to a parental history of MDD.

Overall, (i) having a parental MDD history and (ii) higher levels of neighborhood disadvantage were associated with blunted reactivity to both gain and loss outcome feedback. Importantly, parent history of MDD also moderated the link between the various indices of neighborhood disadvantage and children’s reward outcome processing. Specifically, across all three neighborhood indices, higher levels of neighborhood disadvantage were associated with blunted neural responses (reduced RewP amplitudes) to both gain and loss feedback among children of parents with a history of MDD. In contrast, none of the indices of neighborhood disadvantage were significantly associated with RewP amplitudes among children of never depressed parents. When examining the three domains of childhood opportunity (education, health/environment, and social/economic) separately, the only domain exhibiting significant relations with children’s reward outcome processing was the social/economic domain. Given that the social/economic domain is also the COI index that is most highly correlated with overall neighborhood disadvantage index (i.e., ADI scores), it is possible that this finding was driven by overall neighborhood socioeconomic status rather than specific opportunities/resources. Before drawing strong conclusions about the pattern of findings, we should note the possibility that these findings were influenced by our use of a monetary reward which may more strongly correspond with economic stressors than stressors associated with education or health. Given this, future studies should seek to include other forms of reward processing (e.g., social rewards). As expected, results were somewhat stronger for the PCA-derived RewP-gain/loss than for the mean amplitude-based RewP-gain/loss, which may be because PCA more precisely isolates substages of reward processing, which is thought to increase the signal-to-noise ratio of other overlapping ERP components (Dien, 2012). Finally, regardless of approach, the findings were maintained when accounting for the potential influence of children’s demographic characteristics (age, sex, race/ethnicity), their current depressive symptom level, and family income.

Taken together, these findings suggest that contextual, neighborhood stressors are associated with blunted reactivity to both monetary reward and loss among children, but only among children already at risk due to having a parental history of MDD. In other words, children with a parental MDD history, who are already predisposed to disrupted reward processing, may be more vulnerable to the effects of contextual stress. This is consistent with prior research, which suggests that both parental depression history (see Kujawa & Burkhouse, 2017; Luking et al., 2016) and exposure to stress, especially early life stressors (see Hanson et al., 2021; Novick et al., 2018), may impact neural responses to reward feedback. These findings also extend the recent findings by Granros et al. (2024) in showing that the link between neighborhood disadvantage and reward outcome processing in offspring of parents with a history of MDD generalizes to a different, less urban setting and across different indices of neighborhood disadvantage that encompass measures of neighborhood threat and deprivation. More broadly, this pattern of results can be contextualized by Bronfenbrenner’s bioecological model of human development, which suggests that different environmental systems (e.g., an individual’s home environment, neighborhood, etc.) interact to influence youth’s development (Bronfenbrenner, 1977; Bronfenbrenner & Morris, 2007).

Notably, none of our findings were moderated by feedback type (i.e., gains vs. losses), indicating that children of parents with MDD residing in more disadvantaged neighborhoods experience less reactivity to both positive and negative outcome feedback. This suggests that contextual and familial stressors may impact the overall blunting of feedback cues, regardless of valence. This differs from the Granros et al. (2024) findings, which show that among high-risk youth, neighborhood disadvantage is associated with a reduced differentiation between gains and losses. However, our findings are consistent with the emotion context insensitivity theory, which posits that individuals with, or at risk for, depression exhibit a general disengagement from both positive and negative aspects of their environment (Rottenberg et al., 2005). Thus, children’s blunted reactivity to general feedback could serve as a mechanism of risk linking environmental stress and negative psychological outcomes, like depression. This reduced reactivity to outcome feedback could perhaps be targeted through interventions aimed at bolstering positive affect could be used to increase responsivity to positive outcomes (see Burkhouse et al., 2023; Craske et al., 2019), which could reduce future risk for psychopathology.

This study had various strengths, including the large and representative community sample. Another strength is the inclusion of multiple, objective measures of neighborhood disadvantage, incorporating not only a broad index of disadvantage (ADI) but also more specific indices of threat (crime) and deprivation (opportunity). Despite these strengths, there are some limitations that should be addressed in future research. First, this sample consisted of youth who were in middle to late childhood, which may not capture the developmental peak of reward sensitivity as previous research shows that adolescents are particularly sensitive to rewards (Spear, 2000; Urošević et al., 2012). Thus, future research should explore how these variables relate to reward processing in a slightly older sample and how these relations may change as children age into adolescence. Second, this study focused exclusively on objective measures of neighborhood disadvantage and did not account for subjective measures of disadvantage (e.g., perceived disadvantage compared to peers), which may uniquely relate to reward processing and provides an important avenue for future research. Finally, as noted above, this study only investigated neural responses to one type of reward (monetary gains vs. losses) and did not explore how these variables may relate to social reward, which may be particularly salient during this developmental phase. Therefore, future research should examine whether these findings generalize across different reward stimuli.

In sum, the current findings show that multiple indices of neighborhood disadvantage predict blunted neural responses to monetary rewards and losses in youth, particularly among youth with a parental history of MDD. Given that dysfunctional reward processing is a predictor and correlate of psychopathology (e.g., depression), the findings from this study provide a potential biomarker that may help to identify aspects of youth’s broader environment that contribute to the risk of developing emotional and behavioral disorders. Ultimately, this information can be used to develop and improve individual- and community-level interventions aimed at mitigating risk for psychopathology in youth.

## Data Availability

Data reported in this article are available upon request from the corresponding author.

## Footnotes

1 The terms RewP and feedback negativity (FN) are used inconsistently in prior literature to describe initial neural activity after receiving reward feedback. In this article, we use the term RewP-gain to describe response to gain feedback, RewP-loss to describe response to loss feedback, and RewP-gain/loss to describe response to feedback more broadly (incorporating both gains and loss).

2 Crime risk data were only available for the primary county in which study participants lived. This covered 85% of participants and included 20 different zip codes.

3 We had planned missingness for Crime Risk data because it was only available for one county int this sample, which included 85% of our participants. Therefore, we did not estimate missing values for Crime Risk.

4 The crime risk index also includes separate measures of personal and property crime. Results with these indices mirrored the overall crime risk results where there were no main effects of either type of crime on RewP amplitudes but there was a significant interaction with parental MDD history for both personal crime (*p* =.02) and property crime (*p* =.01) where higher levels of both types of crime were associated with blunted RewP amplitudes but only among children with a parental MDD history. Details of these analyses are available from the first author.

5 Given that some of the predictor variables and covariates are significantly correlated, we added each of the covariates into separate models because adding all covariates in one model led to suppressor effects.

6 As with the PCA analyses, we also ran analyses with personal and property crime indices. We did not find significant main effects of personal or property crime on RewP amplitudes but we found that the parental MDD history × personal crime (*p* =.02) and the parental MDD history × property crime (*p* =.04) interactions were significant and in the same direction as for overall crime risk.
